# Phosphorylation of Human Tristetraprolin in Response to Its Interaction with the Cbl Interacting Protein CIN85

**DOI:** 10.1371/journal.pone.0009588

**Published:** 2010-03-08

**Authors:** Vishram P. Kedar, Martyn K. Darby, Jason G. Williams, Perry J. Blackshear

**Affiliations:** 1 The Laboratory of Signal Transduction, National Institute of Environmental Health Sciences, Research Triangle Park, North Carolina, United States of America; 2 Protein Microcharacterization Core Facility, National Institute of Environmental Health Sciences, Research Triangle Park, North Carolina, United States of America; 3 Departments of Medicine and Biochemistry, Duke University Medical Center, Durham, North Carolina, United States of America; Victor Chang Cardiac Research Institute (VCCRI), Australia

## Abstract

**Background:**

Tristetraprolin (TTP) is the prototype member of a family of CCCH tandem zinc finger proteins and is considered to be an anti-inflammatory protein in mammals. TTP plays a critical role in the decay of tumor necrosis factor alpha (TNF) mRNA, among others, by binding AU-rich RNA elements in the 3′-untranslated regions of this transcript and promoting its deadenylation and degradation.

**Methodology/Principal Findings:**

We used yeast two-hybrid analysis to identify potential protein binding partners for human TTP (hTTP). Various regions of hTTP recovered 31 proteins that fell into 12 categories based on sequence similarities. Among these, the interactions between hTTP and CIN85, cytoplasmic poly (A) binding protein (PABP), nucleolin and heat shock protein 70 were confirmed by co-immunoprecipitation experiments. CIN85 and hTTP co-localized in the cytoplasm of cells as determined by confocal microscopy. CIN85 contains three SH3 domains that specifically bind a unique proline-arginine motif (PXXXPR) found in several CIN85 effectors. We found that the SH3 domains of CIN85 bound to a PXXXPR motif located near the C-terminus of hTTP. Co-expression of CIN85 with hTTP resulted in the increased phosphorylation of hTTP at serine residues in positions 66 and 93, possibly due in part to the demonstrated association of mitogen-activated protein kinase kinase kinase 4 (MEKK4) to both proteins. The presence of CIN85 did not appear to alter hTTP's binding to RNA probes or its stimulated breakdown of TNF mRNA.

**Conclusions/Significance:**

These studies describe interactions between hTTP and nucleolin, cytoplasmic PABP, heat shock protein 70 and CIN85; these interactions were initially discovered by two-hybrid analysis, and confirmed by co-immunoprecipitation. We found that CIN85 binding to a C-terminal motif within hTTP led to the increased phosphorylation of hTTP, possibly through enhanced association with MEKK4. The functional consequences to each of the members of this putative complex remain to be determined.

## Introduction

The cellular response to physiological and environmental stimuli involves regulation of gene expression at multiple levels. Although transcription is a major site of control, post-transcriptional mechanisms also play pivotal roles in regulating gene expression. RNA translation and mRNA degradation are dependent on specific *cis*-acting sequences and *trans*-acting factors [1], [2], and expression of the *trans*-acting regulatory proteins is controlled at multiple levels.

A crucial level of control is exerted at the level of mRNA decay in the case of the pro-inflammatory polypeptide tumor necrosis factor alpha (TNF), one of whose regulators is the CCCH tandem zinc finger protein tristetraprolin (TTP; also known as ZFP36, NUP475 and GOS24) [3]. However, the precise mechanisms by which TTP controls TNF mRNA stability are unclear, with various data supporting roles for the proteasome, the exosome, and RNA processing-bodies (P-bodies). TTP, through its CCCH tandem zinc finger (TZF) domain, first binds to the AU-rich element (ARE) of the TNF transcript with high affinity to a nine base sequence, UUAUUUAUU, that is repeated several times in the TNF mRNA 3′-untranslated region (UTR) [4], [5], [6]. RNA binding is followed by deadenylation and ultimately transcript decay [7], [8], [9], [10], [11], [12], [13]. The other three mammalian TTP family members, ZFP36L1 (also known as TIS11B, BRF1, ERF1, and CMG1) [14], ZFP36L2 (also known as TIS11D, BRF2, and ERF2) [15], and ZFP36L3 [16], [17] share TTP's ability to accelerate the deadenylation and decay of ARE-containing transcripts in cell transfection studies and in cell-free deadenylation assays.

TTP is subject to many modes of regulation, including its agonist-stimulated induction at the transcriptional level, nucleocytoplasmic shuttling, interactions with cellular proteins, and phosphorylation. In an attempt to identify some of the protein binding partners that might influence TTP's activities, we conducted an extensive yeast two-hybrid screen, using full-length human TTP and its fragments as “baits”. Human TTP (hTTP) fragments bound to regions of 31 proteins that fell into 12 categories based on sequence characteristics. One novel hTTP binding protein identified by this technique was the adaptor protein human Cbl-interacting protein CIN85, also known as Ruk, SETA or SH3KBP1 (SH3-domain kinase binding protein 1). CIN85 is known to associate with Cbl [18], Src family kinases [19], p85 phosphatidyl inositol-3 (PI-3) kinase [20] and MEKK4 [21]. The amino-terminus of CIN85 contains three SH3 domains known to mediate protein–protein interactions by binding to unique proline-rich motifs. CIN85 is thought to participate in many important cellular processes including T-cell activation, kidney function, apoptosis in neuronal cells and endocytosis, in part by regulating receptor tyrosine kinase (RTK) signaling [22]. We found that binding of CIN85 to hTTP occurred at a proline-rich motif that was not found in mouse TTP or in the other human TTP family members; surprisingly, this binding led to increased phosphorylation of hTTP at serine residues in positions 66 and 93.

## Results

### Results of Two Hybrid Screen

In an automated two-hybrid screen, full-length hTTP was found to be self-activating; consequently, a series of protein fragments was expressed. In some cases, screens for possible hTTP interacting proteins were performed in the presence of ARE-RNA fragments derived from the TNF mRNA sequence.

Human TTP fragments were screened against three separate activation domain libraries, derived from human spleen, brain, and from a mixture of breast and prostate cancer cell lines. Thirty-one hTTP fragments were fused with the Gal4 DNA binding domain. Of these constructs, eight containing amino-terminal amino acids 1–50 were self-activating; however, eight others, typically containing the TZF domain, were successful in recovering interacting proteins. The amino acid sequences of these interactors were analyzed for identification as described [23]. In total, 31 “prey” interactors were identified that comprised multiple fragments identified in several separate screens. These potential interactors fell into 12 different protein categories based on specific motifs in their sequences. A list of the potential interactors focused on in this paper is shown in Table 1. These proteins included other TTP family members, several types of RNA binding proteins, RNA helicases, the CCR4-NOT1 deadenylase, and proline-rich domain containing proteins.

**Table 1:** Potential protein interactors with hTTP.

hTTP (aa #)	Library (human)	Interactor (Accession #)	Interactor (aa #)	Function
150–326, 223–326.	Brain/Spleen	CIN85 (NP_114098.1)	4–264, 4–450.	CBL-interacting protein: Endocytosis, signaling, apoptosis. etc.
90–180	Breast/Prostate cancer	Nucleolin (NP_0053722)	375–692	rDNA transcription, rRNA maturation, ribosome assembly and nucleocytoplasmic transport.
90–180	Spleen	PABPC1 (NP_002559)	100–309, 236–399, 99–422, 178–466, 79–400, 95–408.	Translational initiation, mRNA stabilization and nucleocytoplamic shuttling.
90–180	Breast/Prostate cancer	PABPC1 (NP_002559)	1–246, 187–482.	Translational initiation, mRNA stabilization and nucleocytoplasmic shuttling.
90–180	Spleen	HSP70 (NP_005337.2)	11–416	Participates in ubiquitin- proteasome pathways.

Amino acid (aa) residue numbers for hTTP were from GenBank RefSeq NP_003398.1. The aa residues shown for potential hTTP-interacting proteins are from the RefSeq numbers listed in the “Interactors” column in the table.

### Co-Immunoprecipitation of hTTP with Potential Interacting Proteins

**Figure 1:**
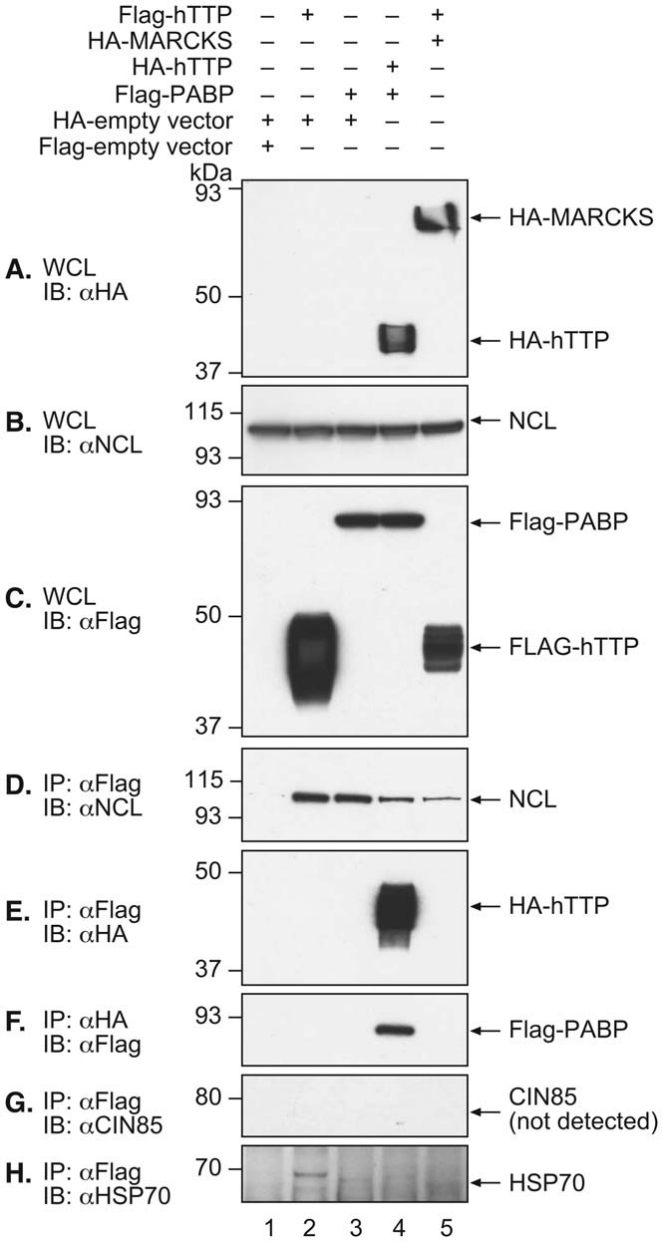
Co-immunoprecipitation of hTTP with potential interacting partners. In this and subsequent figures, extracts were prepared in RIPA buffer from HEK 293 cells transfected with DNA encoding the HA- and Flag-tagged expression vectors indicated at the top of each gel lane by the “+” sign. Total DNA transfected was 5.0 µg per 100 mm petri dish. For each western blot shown, the immunoprecipitating antibody (IP) and the subsequent immunoblotting antibody (IB) are indicated to the left of each panel, as are the positions of protein molecular weight standards. The immunoreactive protein species are indicated by the labeled arrows to the right of each blot. Each immunoprecipitation used 1 mg of cellular lysate protein as the starting material. In some cases, the blots are of whole cell lysates (WCL) (50 µg of total protein per lane) instead of from immunoprecipitations to confirm expression of the respective protein in the lysates prior to immunoprecipitation. In addition to the epitope-tag antibodies indicated, western blotting in this case also used antibodies to endogenous nucleolin (NCL), CIN85, and HSP70. See the Results section for additional details.

We selected four potential TTP binding partners, cytosolic poly (A) binding protein (PABP/PABPC1), nucleolin, heat-shock protein 70 (HSP70), and CBL-interacting protein 85 (CIN85) (Table 1), for further validation using co-immunoprecipitations between hTTP and epitope-tagged candidate proteins in HEK 293 cells. Endogenous, untagged nucleolin co-immunoprecipitated with Flag-tagged or HA-tagged hTTP (Fig. 1D, lanes 2, 4 and 5), but not with extracts from cells transfected with plasmids encoding HA- and Flag-tagged empty vectors (Fig. 1D, lane 1). We also tested nucleolin binding to cytoplasmic PABP, which we also identified as a potential interactor with hTTP (Table 1) and confirmed by co-immunoprecipitation in the present study (Fig. 1F, lane 4). Nucleolin was pulled down both by hTTP alone and by PABP alone when they were each immunoprecipitated with anti-Flag antibody (Fig. 1D, lanes 2 and 3), and by pulldown of hTTP when it was co-expressed with PABP (Fig. 1D, lane 4). These results suggest that nucleolin can form complexes with hTTP and PABP individually, as well as with the complex formed when they are expressed together. The hTTP did not bind to the negative control protein, human MARCKS (Fig. 1F, lane 5).

The association between Flag-hTTP and HSP70 was confirmed when the cell lysate was immunoprecipitated using anti-Flag antibody and probed with an anti-HSP70 antibody (Fig. 1H, lane 2), confirming HSP70 as a binding partner of hTTP under these conditions. However, although CIN85 was identified in the two hybrid screen as a potential interactor with hTTP, the same anti-Flag immunoprecipitation did not pull down detectable endogenous CIN85 (Fig. 1G, lane 2), prompting further investigation using overexpression of CIN85 protein. We have been unable to detect endogenous CIN85 expression in HEK 293 cells, either by western blotting, using CIN85 antibody HQ-17 (Sigma) (data not shown) or by northern blotting (see below).

### Interaction of Human TTP and CIN85

**Figure 2:**
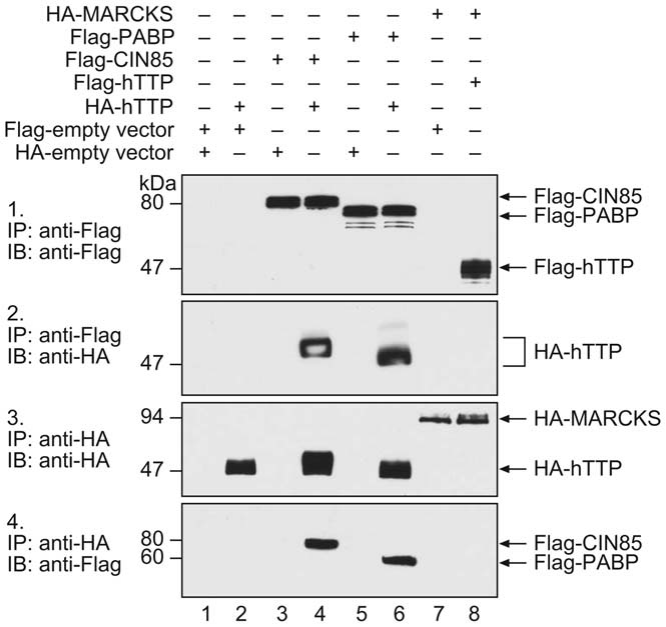
Interaction of TTP and with PABP and CIN85. Abbreviations and other details are as described in the legend to Fig. 1.

We investigated a possible association between these two proteins by transfection and co-immunoprecipitation (Fig. 2A). The association of hTTP with PABP was used as a positive control, with the MARCKS protein used as negative control. Binding of hTTP to CIN85 (Fig. 2A2, lane 4) and to PABP (Fig. 2A2, lane 6) was readily detected in anti-Flag immunoprecipitations. Unexpectedly, the migration of hTTP appeared to be retarded after co-immunoprecipitation with CIN85 (Fig. 2A2, compare lanes 4 and 6), raising the possibility that there might be increased phosphorylation of hTTP under these conditions. When the co-immunoprecipitation was performed instead with the anti-HA antibody followed by immunoblotting with the anti-Flag antibody, both CIN85 and PABP were pulled down as hTTP binding partners (Fig. 2A4, lanes 4 and 6). There was no apparent binding of TTP to MARCKS (Fig. 2A2 and 2A4, lane 8). These results confirmed that both PABP and CIN85 could interact with hTTP under these experimental conditions, and that the migration of hTTP was apparently retarded after it had been co-expressed with CIN85.

### Association of CIN85 with Other TTP Family Members

**Figure 3:**
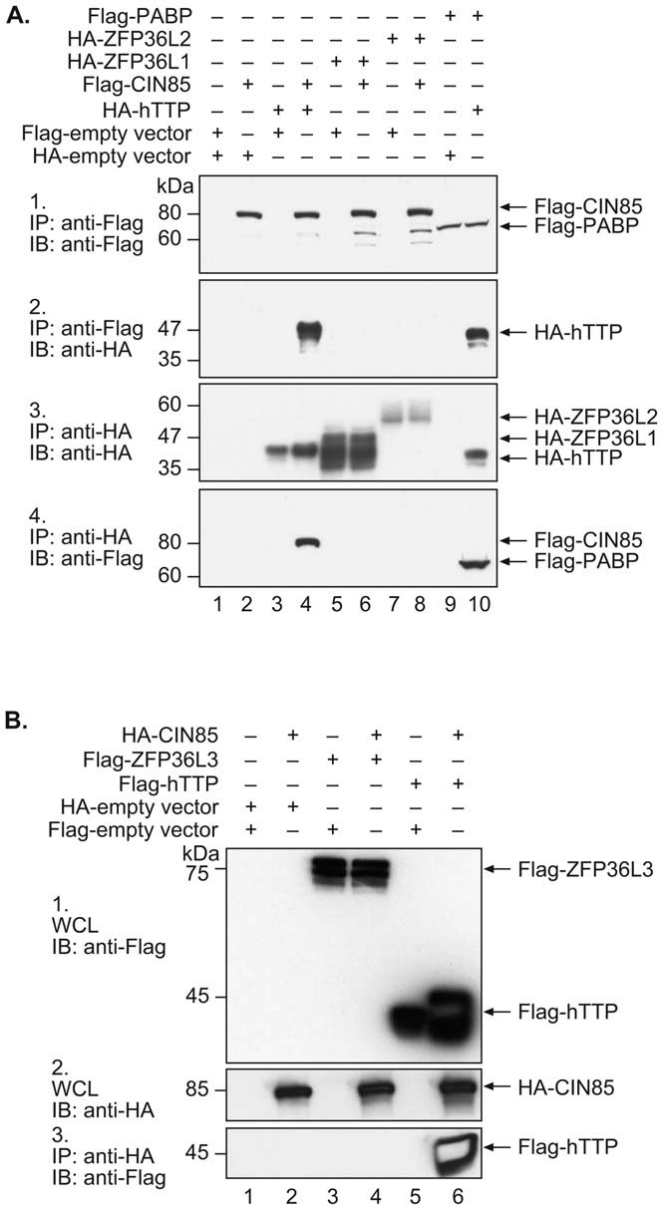
Association of CIN85 with hTTP family members. Abbreviations and other details are as described in the legend to Fig. 1, with the exception of Panel 3B1, which shows an immunoblot of the whole cell lystate probed with anti-Flag antibody.

We also did not identify CIN85 in other two-hybrid experiments with human ZFP36L1, human ZFP36L2, or mouse ZFP36L3 (data not shown). We therefore co-expressed CIN85 with each of these full-length TTP family members in HEK 293 cells and attempted to co-immunoprecipitate these proteins from lysates expressing both pairs of proteins. Again, hTTP could be brought down by CIN85 (Fig. 3A2, lane 4) and by PABP (Fig. 3A2, lane 10) but CIN85 did not pull down hZFP36L1 (Fig. 3A2, lane 6), hZFP36L2 (Fig. 3A2, lane 8) or mouse ZFP36L3 (Fig. 3B3, lane 4). Thus, we found no evidence of an association between CIN85 and the other human TTP family members ZFP36L1 or ZFP36L2, or between CIN85 and either mouse ZFP36L3 or TTP. Once again, CIN85 was able to pull down hTTP as a positive control (Fig. 3B3, lane 6).

### Sites of Interaction in hTTP and CIN85

**Figure 4:**
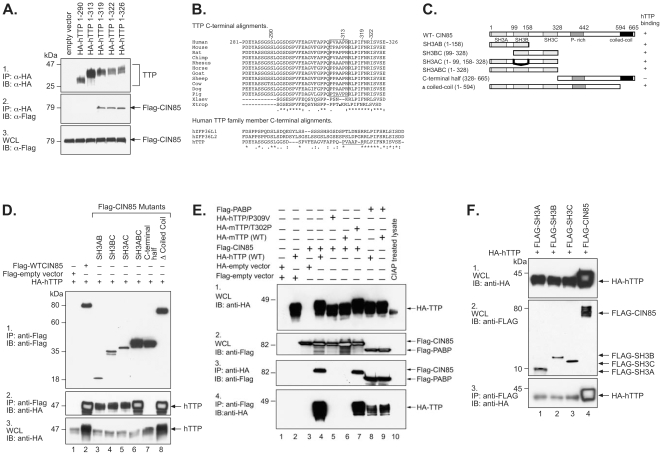
Sites of interaction in hTTP and CIN85. In A, Flag-CIN85 was co-expressed with various C-terminal truncated forms of HA-hTTP, as indicated at the top of the figure. Abbreviations and other details are as described in the legend to Fig. 1. In B is shown a ClustalW alignment of the putative PXXXPR CIN85 binding motif near the C-terminus of hTTP aligned with the C-termini of TTP from various vertebrate species, as well as with the TTP family members ZFP36L1 and ZFP36L2 from human. The sequences shown are derived from the following GenBank accession numbers: human TTP (NP_003398.1), mouse (NP_035886.1), rat (NP_579824.2), chimpanzee (XP_001136016), rhesus (XP_001086084.1), horse (CD536523.1), sheep (NP_001009765.1), cow (NP_776918.1), dog (XP_541624.2), pig (DY419026), *Xenopus tropicalis* (Xtrop) (NP_001106542.1) and *Xenopus laevis* (Xlaev) (NP_001081884.1) The putative CIN85 binding PXXXPR motif in human and other mammalian TTPs (but not mouse) is boxed. This motif is also not present in the orthologues from the two frog species. The typical PXXXPR motif is also not present in the in the C-termini of the other human TTP family members ZFP36L1 (NP_004917) and ZFP36L2 (NP_008818). In C are shown schematic representations of full-length CIN85 and its truncations. The names of various truncations of CIN85 and their amino acid positions are indicated on the left. Positions of the Src homology domains 3 (SH3) A, B and C) are indicated in light grey boxes; the proline rich region (P- rich) is shown as a darker grey box; and the C-terminal coiled-coil region is shown as a black box. The ability of each construct to bind hTTP is indicated on the right. The data supporting this diagram are shown in D and F, with the transfected plasmids shown at the top of the blot, and with other aspects of the western blots as described in the legend to Fig. 1. Panel 3 documents the expression of full-length hTTP in each WCL (50 µg/lane). In E are shown data from mutations in the **P**XXXPR motif in hTTP, and the corresponding sequence in mouse TTP. Abbreviations and other details are as in the legend to Fig. 1.

Since in our two hybrid screen two separate hTTP baits containing C-terminal hTTP sequences identified multiple clones representing two fragments of human CIN85 (aa 4–264 and aa 40–458), we predicted that the C-terminal region of hTTP was involved in its binding to the N-terminus of CIN85. The extreme C-terminus of hTTP contains a potential CIN85 binding site, represented by the consensus PXXXPR [21], [24], [25]. We therefore tested a series of C-terminal deletion mutants of hTTP against full-length CIN85 in co-immunoprecipitation assays. The names of these constructs are indicated at the top of the gel lane in Fig. 4A. We found that removal of five or eight C-terminal amino acids had no effect on CIN85 binding (Fig. 4A2, lanes 4–6); however, removal of either 14 (aa 1–313, Fig. 4A2, lane 3) or 37 (aa 1–290, Fig. 4A2, lane 2) C-terminal amino acids in hTTP, in both cases removing the PXXXPR motif, completely eliminated the binding, suggesting that this PXXXPR motif is critical for forming the complex with CIN85. Equivalent expression of the various mutant forms of hTTP is documented in Fig. 4A1 (lanes 2–6).

In a separate two hybrid screen conducted using fragments of mouse TTP (mTTP) as baits, we did not identify CIN85 as an mTTP interactor (data not shown). Mouse TTP also failed to bind CIN85 in the co-immunoprecipitation assay (Fig. 4E, lane 6). Since CIN85 did not bind to mouse TTP or to the other human TTP family members (Fig. 3A and B), we aligned the C-terminal sequences of these proteins, and found that the intact PXXXPR domain did not occur in mouse TTP, but did occur in all other mammals for which sequence was available (Fig. 4B). That this was not an artifact of a mouse sequencing error was confirmed by alignment of the mouse C-terminal protein sequence with ESTs in GenBank, which demonstrated at least nine mouse ESTs with identical translated sequence to that shown in Fig. 4B. We also could not find intact PXXXPR motifs elsewhere in the mouse protein.

We next attempted to define which region of CIN85 was responsible for binding to hTTP. In its N-terminal region, CIN85 contains three Src homology 3 (SH3) domains, SH3A, SH3B, and SH3C. The region around amino acid 400 is proline rich, and the extreme C-terminus contains a coiled-coil domain. The details of a series of Flag-tagged expression constructs in which some of these domains were deleted are summarized schematically in Fig. 4C. Each of these deletion constructs was then separately co-expressed with full length HA-tagged hTTP. The results from immunoprecipitations performed on cell lysates expressing these protein fragments are summarized in Fig. 4D and 4F. These data demonstrated that hTTP could bind to all of the N-terminal CIN85 fragments carrying various combinations of the three SH3 domains (Fig. 4D2, lanes 2–6, 8), and to individual SH3 domains (Fig. 4F, lanes 1–3), but not to the C-terminal half of CIN85 that lacked any of the SH3 domains (Fig. 4D2, lane 7). These results suggest that the binding of CIN85 to hTTP is mediated by the interaction of the SH3 domains of CIN85 to the PXXXPR motif located within the C-terminus of hTTP.

In order to further test whether the first proline in the PXXXPR motif of hTTP is necessary for its binding to CIN85, we changed the first proline in this motif to valine (P309V). We also replaced the corresponding threonine with proline (T302P) in mTTP to recreate the human PXXXPR motif. These expression plasmids were then transfected into HEK293 cells, and co-immunopreciptations on the resulting lysates were performed using antibodies against HA or Flag, as described above. As expected, WT hTTP and CIN85 were co-immunoprecipitated by anti-HA or anti-Flag antibodies (Fig. 4E3 and 4E4, lane 4 in each case), whereas WT mTTP did not bring down CIN85 (Fig. 4E3, lane 6). Mutating the first P in the human PXXXPR motif (P309V) eliminated the binding of hTTP to CIN85 (Fig. 4E3, lane). Creation of the PXXXPR motif in mTTP (T302P) permitted the binding of mTTP to CIN85 (Fig. 4E3, lane 7). The roughly equivalent expression of the mutant proteins was demonstrated in Fig. 4E1. PABP was found to associate with both hTTP and mTTP when it was tested as a positive control (Fig. 4E3, lanes 8 and 9, respectively). Neither hTTP nor CIN85 was co-immunoprecipitated by empty vector negative controls (Fig. 4E3, lanes 2 and 3). As before, hTTP expressed in the presence of CIN85 was found to migrate more slowly than hTTP not expressed with CIN85 (Fig. 4E1, compare lanes 2 and 4). This retarded migration disappeared in the case of the non-binding TTP mutant P309V (Fig. 4E1, lane 5), but was present in the newly binding-competent mouse T302P mutant (Fig. 4E1, lane 7). When the hTTP in the lysates was dephosphorylated with CIAP, the hTTP protein migrated to a position roughly corresponding to its predicted size of approximately M_r_ 34,000 (Fig. 4E1, lane 10).

These results demonstrate that the PXXXPR motif is responsible for hTTP's binding to CIN85, and that the presence of this intact motif in either human or mouse TTP results in its retarded migration in SDS gels after co-expression of CIN85.

### Human TTP Forms a Complex with MEKK4 and CIN85

**Figure 5:**
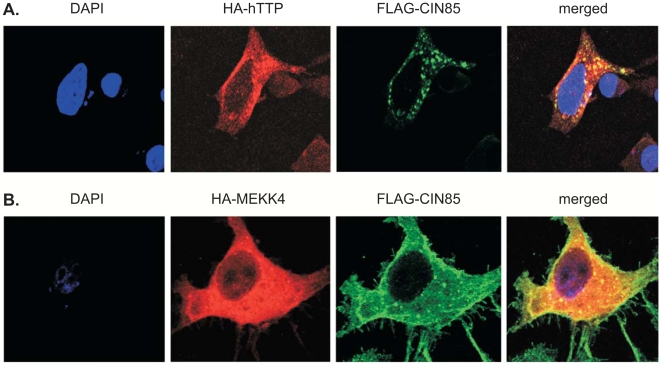
Localization of hTTP and MEKK4 with CIN85 in cultured cells. In A, HEK 293 cells were transfected with plasmids for the expression of HA-hTTP (red) and Flag-CIN85 (Green), or (B) HA-MEKK4 (red) and Flag-CIN85 (green). The cells were stained with primary antibodies, either an anti-HA polyclonal or anti-Flag monoclonal, followed by the secondary antibodies Alexa 594 anti-rabbit (red) or Alexa 488 anti-mouse (green), respectively. Nuclei (blue) were stained with DAPI. The cells were visualized and images obtained by confocal microscopy. The merged images of two protein signals, indicating areas of apparent co-localization, are shown in yellow.

**Figure 6:**
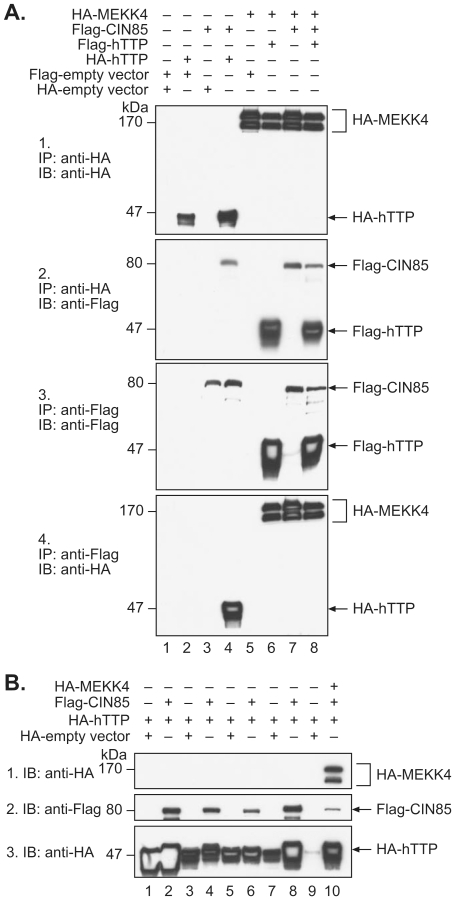
Interaction between hTTP and both MEKK4 and CIN85. In A, tagged expression constructs of MEKK4, CIN85 and hTTP were co-transfected in pairs (lanes 1 to 7) or together (lane 8) with or without empty vectors. Abbreviations and other details are as described in the legend to Fig. 1. In B are shown whole cell lysates, demonstrating the protein expression from the various expression plasmids or empty vectors, as well as the apparent shift in the M_r_ of hTTP when co-expressed with CIN85.

Because of the possible increase in hTTP phosphorylation that occurred upon co-expression with CIN85, we evaluated the possibility that mitogen-activated protein kinase kinase kinase 4 (NP_005913.2; synonyms: MEKK4 kinase, MAP3K4; FLJ42439; PRO0412; KIAA0213; MAPKKK4; MTK1; JNK/p38 MAP kinase kinase kinase MEKK4), a known binding partner and effecter of CIN85 [18], [21], could form a complex with hTTP in the presence or absence of CIN85. Confocal microscopy of HEK 293 cells transfected with plasmids expressing hTTP, CIN85, and MEKK4 proteins showed that hTTP and CIN85 were at least partially co-localized in the cytoplasm (Fig. 5A), as were CIN85 and MEKK4 (Fig. 5B). We next tested the possibility of associations among the proteins by co-immunoprecipitation. When an HA-MEKK4 immunoprecipitation was blotted with an anti-Flag antibody, then Flag-hTTP was readily detected (Fig. 6A2, lane 6), demonstrating that that hTTP could associate with MEKK4 directly. These results were supported by the ability of Flag-hTTP to bring down HA-MEKK4 (Fig. 6A4, lane 6). An additional experiment demonstrated that HA-MEKK4 brought down Flag-labeled wild-type hTTP, as well as the T302P mutant form of hTTP (data not shown). As expected from previous results in the literature [21], Flag-CIN85 was found to co-immunoprecipitate with HA-MEKK4 (Fig. 6A2, lane 7; Fig. 6A4, lane 7). When all three proteins were expressed together, anti-HA-MEKK4 could bring down both Flag-CIN85 and Flag-hTTP (Fig. 6A2, lane 7), and Flag-hTTP and CIN85 could bring down HA-MEKK4 (Fig. 6A4, lane 7). These results demonstrate that both hTTP and MEKK4 can associate with CIN85, and hTTP can associate with MEKK4, in separate two-protein complexes; in addition, the three proteins appear to be able to form three-protein complexes with each other. As before, none of these proteins was found to bind non-specifically when co-expressed with empty vectors (Fig. 6A2, lane 2; Fig. 6A4, lane 3).

### Co-Expression with CIN85 Results in Retarded Migration of hTTP

TTP is known to be heavily phosphorylated [8], [10], [26], [27], [28], [29], [30], [31], [32], [33], [34]. In previous experiments, we observed that TTP expressed in HEK293 cells appears on SDS gels as multiple bands of approximately M_r_ 47,000, which collapses with phosphatase treatment to its predicted size of M_r_ 34,000 [26], [27], [28], [35]. As shown above, when hTTP was co-expressed with CIN85, the hTTP protein had an apparently greater M_r_ than the protein expressed alone, or expressed with another binding partner such as PABP (Fig. 2A2, lanes 4 and 6, Fig. 4D3, lane 2, Fig. 4E1, lanes 4 and 7, and Fig. 6A1, lane 4), suggesting that hTTP is hyper-phosphorylated in response to its interaction with CIN85. This result was seen in many independent experiments (Fig. 6B3, lanes 2, 4 and 8). Similar retarded migration was seen when hTTP and CIN85 were co-expressed with MEKK4 (Fig. 6B3, lane 10).

### Co-Expression of CIN85 with hTTP Results in Increased Phosphorylation of hTTP at Serine Residues 66 and 93

**Figure 7:**
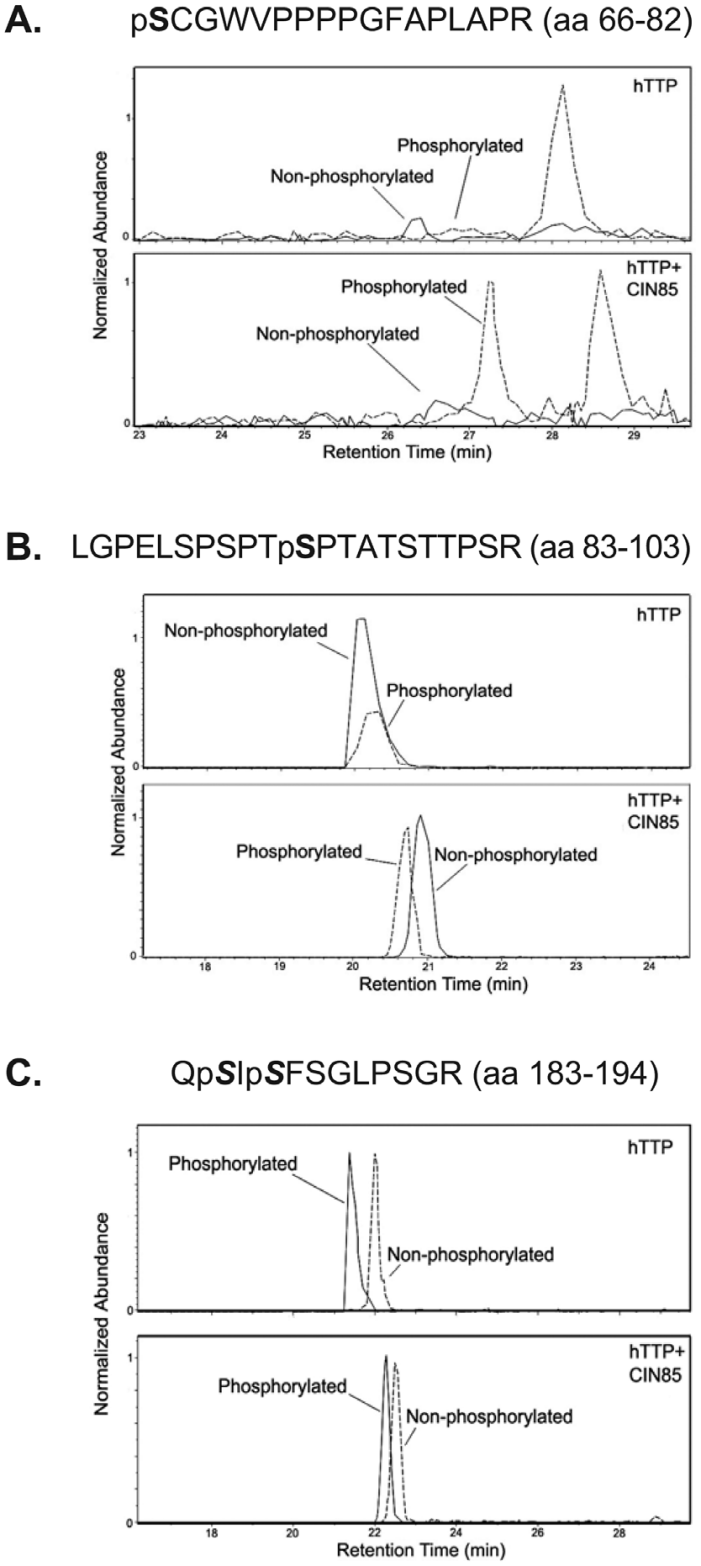
Phosphorylation sites in hTTP co-expressed with CIN85. Panels A, B, and C are extracted ion chromatograms (EICs) for residues 66–82 (A), 83–103 (B), and 183–194 (C) generated from nanoLC-ESI-MS runs derived from tandem MS data of ions m/z 915.4, m/z 1083.5, and m/z 658.5, corresponding to the phosphorylated peptides 66–82, 83–103, and 183–194, respectively (Data/Fig. not shown). The normalized responses demonstrated in the EICs are an estimation of the abundance of the ion of interest. The currents from the non-phosphorylated peptides are represented as solid lines, and the ion currents attributed to the phosphorylated forms of the same peptides are shown as dashed lines. Duplicate technical replicates yielded similar results.

Since the co-expression of hTTP with CIN85 produced an increase in the apparent M_r_ of hTTP on SDS/PAGE, suggesting increased phosphorylation, we analyzed the phosphorylation state of hTTP using FLAG-affinity-purified protein from HEK 293 cells expressing FLAG-hTTP alone or Flag-hTTP and HA-CIN85 together. Analyses for phosphopeptide identification were performed using a variety of mass spectrometric, affinity and chromatographic techniques including ESI-MS and MS/MS as well as metal oxide affinity chromatography and reverse-phase liquid chromatography. After phosphopeptides were identified, LC-ESI-MS runs of hTTP digests from cells with and without co-expression of CIN85 were performed in efforts to estimate relative amounts of phosphorylation at each of the identified sites. We identified two phosphopeptides, **S**CGWVPPPPGFAPLAPR and LGPELSPSPT**S**PTATSTTPSR (serines in bold type represent the phosphorylated residues), in which the site of phosphorylation could be unambiguously assigned to Ser 66 and Ser 93, respectively. We identified a third phosphopeptide, Q*S*I*S*FSGLPSGR (serines in italics represent the residues at which the phosphorylation could be occurring), that contained a single phosphorylation, but we could not determine whether the phosphorylation occurred on Ser 184 or Ser 186. Overall coverage of the protein was approximately 50% for the hTTP-only sample, and ∼40% for the hTTP plus CIN85 sample. Phosphorylation at Ser 66 was increased several-fold in the presence of CIN85 (Fig. 7A) and moderately at Ser 93 (Fig. 7B). The additional site at either Ser 184 or Ser 186 did not appear to be affected by the co-expression of CIN85 (Fig. 7C). We previously identified Ser 66 and Ser 93 as potential phosphorylation sites for PKCμ and p38 protein kinase, respectively, as predicted by (http://scansite.mit.edu) (Cao et al., 2007). Evaluation of these and the other sites by a variety of programs (http://bioinformatics.lcd-ustc.org/PPSP/; 22 http://www.cbs.dtu.dk/services/NetPhosK/; http://scansite.mit.edu/) suggested that Ser 66 could in addition be a site for MAPKAP kinase 2, and that Ser 93 could also be a site for cyclin-dependent kinase 5.

Although the extent of phosphorylation in the peptide QSISFSGLPSGR does not appear to change in response to CIN85 co-expression, to our knowledge neither Ser 184 nor Ser 186 has been previously characterized as phosphorylated. The same predictive programs used for Ser 66 and Ser 93 suggest that phosphorylation of Ser 186 could represent a site for AMP-dependent kinase, calcium calmodulin dependent kinases, MAPKAP kinase 2 and MAP kinase kinase kinase. Meanwhile, Ser 184 is predicted to be a potential site for MAP kinase kinase kinase, protein kinase A, and various protein kinase C isoforms.

### Effect of CIN85 on hTTP Binding to an RNA Probe

**Figure 8:**
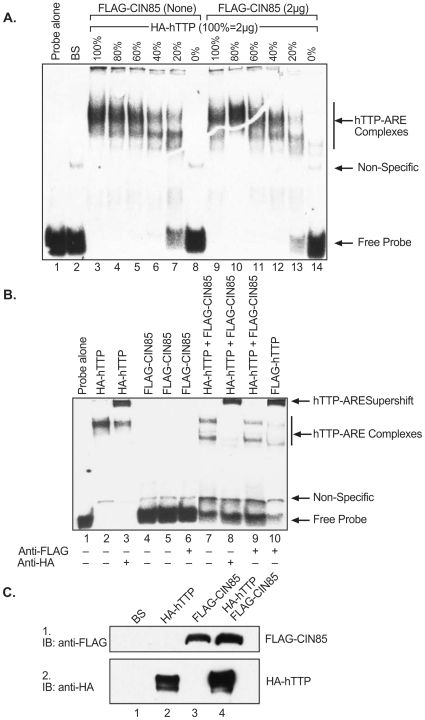
Effect of co-expressed CIN85 on hTTP binding to an RNA probe. Cytosolic extracts of HEK293 cells transfected with vector alone (BS), or vectors expressing HA-hTTP alone and Flag-CIN85 alone, were used in RNA gel shift analysis, using a 5′ biotin-labeled TNF-ARE based RNA probe. In A, protein extracts of containing decreasing amounts of HA-hTTP incubated in the presence or absence of a constant concentration of FLAG-CIN85 were incubated with 0.6 ng of the RNA probe. The migration positions of the hTTP-ARE complexes, the non-specific complexes seen in the HEK 293 cell extract alone, and the RNA probe alone, are all indicated with arrows to the right of Fig. 8A. In B, 2 µg of cellular protein from FLAG-CIN85 expressing cell extracts was incubated with or without HA-hTTP or FLAG-hTTP (1.6 µg) in the presence or absence of the respective epitope tag antibodies. Arrows to the right of the panel are the same as in A, except for the addition of an arrow pointing to the hTTP supershifts. In C, immunoblots were performed using 10 µg of cellular protein from the same extracts, demonstrating expression of the epitope-tagged proteins.

The RNA binding activity of hTTP is essential for initiation of mRNA decay. To determine whether there was any effect of the interaction between CIN85 and hTTP on RNA binding of hTTP, we performed RNA gel shifts using cytosolic extracts containing varying concentrations of hTTP alone or together with CIN85, using a TNF-based ARE RNA probe containing four clustered UUAUUUAUU motifs (residues 1309–1332, GenBank accession number NM_013693.2) [8]. Incubation of this probe with a cytosolic extract prepared from HEK 293 cells transfected with vector alone (BS) revealed the formation of at least one faint band representing interactions of the RNA probe with an endogenous 293 cell protein (Fig. 8A, lane 2; arrow points to the non-specific complex). An extract from cells transfected with the HA-hTTP expression construct was diluted with an otherwise identical extract from cells transfected with vector alone that contained the identical concentration of protein; the percentage of this combined extract that contained the TTP-containing extract is indicated at the top of the Fig. 8A. A sample containing 100% hTTP-expressing lysate produced a single, broad, dark hTTP-ARE complex (Fig. 8, lane 3). Lysates expressing decreasing proportions of hTTP exhibited less binding overall, with at least two hTTP-ARE complexes becoming visible (lanes 5–7). The identical experiment was repeated in the presence of a constant amount of CIN85 (Fig. 8A, lanes 9–14). CIN85 alone did not appear to bind significantly to this probe (Fig. 8A, lane 14), nor did it appear to alter the binding of the various concentrations of hTTP (Fig. 8A, lanes 9–11). We confirmed that these complexes contained HA-hTTP by supershift analysis with the anti-HA and anti FLAG antibodies (Fig. 8B). Flag-CIN85 alone did not form a complex with the ARE probe (Fig. 8B, lane 4), as confirmed by the absence of a supershift when the same extract was incubated with the anti-FLAG antibody (Fig. 8B, lane 6). In extracts containing both HA-hTTP and Flag-CIN85, the anti-HA antibody caused a supershift of the hTTP-probe complex (Fig. 8B, lane 8), whereas the anti-FLAG antibody did not cause a supershift (Fig. 8B, lane 9). Fig. 8C documents the immunoreactive expression of both epitope-tagged proteins; each lane contained five times more hTTP and CIN85 as in Fig. 8A (lane 3 for hTTP alone), Fig. 8A, lane 9 (hTTP and CIN85 together), or Fig. 8A, lane 14 (CIN85 alone).

### Co-Expression of CIN85 with hTTP Does Not Affect Destabilization of TNF mRNA

**Figure 9:**
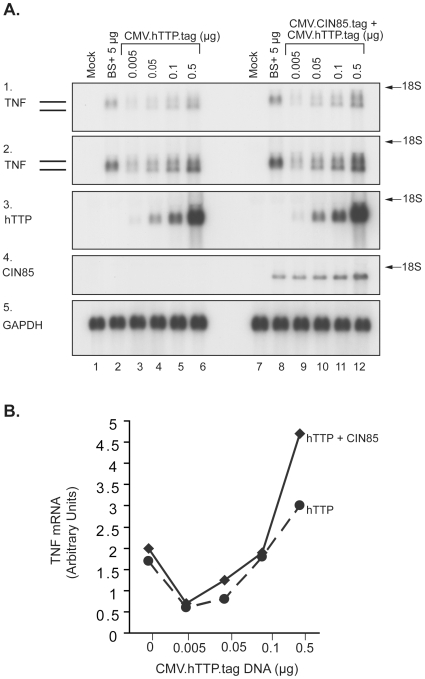
Effects of co-expression of CIN85 on hTTP-promoted destabilization of a TNF mRNA. In A, a CMV-driven mouse TNF-encoding plasmid was co-transfected into HEK 293 cells (lanes 2–6, 8–12) with either vector alone or with the indicated amounts of an hTTP expression construct in the presence or absence of 2 µg of a CIN85 expression construct. Total cellular RNA was harvested 24 h later, and used for northern blotting. Each lane was loaded with 10 µg of total RNA. Lanes 1 and 7 were from mock-transfected HEK 293 cells. Lanes 2 and 8 were from cells transfected with vector alone (BS+; 5 µg/plate). Lanes 3–6, were from cells co-transfected with CMV.mTNF (1 µg) and CMV.hTTP.tag (0.005, 0.05, 0.1, and 0.5 µg/plate, respectively). Lanes 9–12 were from cells co-transfected with CMV.mTNF (1 µg) and CMV.CIN85.tag (2 µg) and CMV.hTTP.tag (0.005, 0.05, 0.1 and 0.5 µg/plate, respectively). Vector was also added as needed to make the total amount of co-transfected plasmids 5 µg/plate in each case. As indicated, the northern blots were probed with either a ^32^P-labeled mTNF cDNA probe (panels 1 and 2, duplicate experiments), an hTTP probe (panel 3), a CIN85 probe (panel 4) or a GAPDH probe (panel 5). Film exposure was 4 h and 7 h, respectively, for panels, A1 and A2 for filters hybridized with an mTNF probe. All other filters were exposed to films for 7 h. The two parallel lines labeled TNF indicate the two species of TNF mRNA discussed in the text. The positions of the 18S rRNA are indicated. In B are shown the phosphorimager values for both species of TNF mRNA as a function of various TTP plasmid amounts, transfected with or without the CIN85 vector. The graph in B is from a single experiment, but is representative of three similar experiments.

To determine whether co-expression of CIN85 with hTTP affected the latter's ability to promote destabilization of a TNF-based RNA transcript, we used a co-transfection assay described previously [8]. When cells were transfected with a TNF-based “target” expression plasmid alone, the transcript was detected as a single band (Figs. 9A1 and 9A2, lanes 2 and 8). When a low concentration of transfected hTTP plasmid DNA was used (5 ng per plate; Figs. 9A1 and 9A2, lane 3), TNF mRNA accumulation was decreased to ∼10 to 20% of that of control, as quantified by Phosphorimager scanning. This decrease in mRNA levels was accompanied by the appearance of a smaller species of mRNA transcript, which first became apparent at 5 ng of DNA (Figs. 9A1 and 9A2, lane 3) but was more evident at 50 ng (Figs. 9A1 and 9A2, lane 4). Most of the TNF transcript was in this smaller form, considered to be the deadenylated form, with increasing amounts of transfected hTTP DNA, beginning at 50 ng (Figs. 9A1 and 9A2, lane 4) through all higher concentrations used (Figs. 9A1 and 9A2, lanes 5 and 6). However, as shown previously, the total TNF transcript accumulation increased substantially at higher concentrations of DNA to reach a maximum of around 200% (Fig. 9B) of that of control at 500 ng of transfected hTTP DNA, a phenomenon we have attributed to protection of the deadenylated species of RNA by high concentrations of TTP [8]. This is considered to be a non-physiological artifact from high TTP concentrations. When the hTTP expression plasmid was co-transfected with the expression plasmid for CIN85, there was essentially no effect on this dose-response curve (Figs. 9A1 and 9A2, lanes 8 to 12). Endogenous CIN85 mRNA was undetectable by this northern analysis (Fig. 9A4, left panel, lanes 1–6). The phosphorimager values from the TNF mRNA northern blot shown in Fig. 9A2 are graphically shown in Fig. 9B. The expression of hTTP and CIN85 mRNAs is shown in Figs. 9A3 and 9A4, as well as the expression of the endogenous GAPDH mRNA (Fig. 9A5) used as a gel loading control.

## Discussion

The anti-inflammatory and cytokine mRNA regulator TTP is an immediate-early gene that is induced by stimuli such as growth factors and mitogens [36], [37]. It is an mRNA binding protein that can promote rapid degradation of mRNA by first binding to AU-rich elements in the mRNA, often in the 3′-untranslated region, and then stimulating deadenylation and ultimately the decay of the target transcript [3], [38], [39], [40], [41], [42], [43]. TTP activity can be regulated at many levels: gene expression; mRNA stability; nucleocytoplasmic shuttling; phosphorylation; protein binding; and others. Both TTP and the pro-inflammatory cytokine TNF can be induced in macrophages by environmental stimuli such as lipopolysaccharides, and by TNF itself; TTP can, in turn, promote the downregulation of TNF by directly targeting its transcript [40].

TTP has long been known to be phosphorylated, in some cases in response to external stimuli; this modification has been shown in some reports to impair its ability to stimulate mRNA decay [28], [30], [31], [32], [44], [45], [46], [47]. TTP activity is also thought to be regulated by binding proteins, including Ccr4, Dcp, Xrn1 [48], MK2 [31], 14-3-3 [49], RISC components Ago/eiF2C [50], nuclear pore protein Nup214 [51] and PP2A [33]. However, a thorough knowledge of TTP's protein binding partners will be important for a complete understanding of its physiological regulation.

We have begun to identify potential TTP-interacting proteins using a series of yeast two-hybrid screens. As described here, this method uncovered direct physical interactions between human TTP (hTTP) and CIN85, the cytoplasmic polyA-binding protein PABP (PABPC1), nucleolin, and HSP70, in addition to 27 other proteins belonging to various protein classes. Since the N-terminal region of hTTP was self-activating in the two-hybrid system, most interactors were recovered with bait peptides encompassing hTTP's TZF domain and its C-terminal proline-rich region. The interactors were recovered from three separate screens using three different activation domain libraries, derived from human spleen, human brain, and from a mixture of human breast and prostate cancer cell lines. As reported here, CIN85 was recovered by binding to C-terminal fragments of hTTP, whereas the TZF domain identified cytosolic PABP, HSP70 and nucleolin.

Although the focus of this paper is on the interaction between TTP and CIN85, the other interactions validated may be of physiological importance. Nucleolin is a major nucleolar protein involved in the regulation of ribosome biogenesis, control of organization of nucleolar chromatin, nucleogenesis [52] and nucleocytoplasmic transport [53]. Its significance as a binding partner of TTP is not clear, although TTP is known to be a nucleocytoplasmic shuttling protein [54], [55], [56]. HSP70 and its family members mediate the folding of newly translated proteins in the cytosol and organelles [57], [58] and also have been reported to promote ARE-mediated mRNA decay [59], [60]. The heat-shock proteins can stimulate the production of certain cytokines (TNF, IL-1, IL-6 and IL-12) and have been reported to possess potent immunoregulatory functions [61], [62].

The PABP proteins comprise a small nuclear isoform and a conserved set of at least three functional proteins: Cytosolic PABP (PABP1 or PABPC1), inducible PABP (iPABP, or PABPC4), and PABP3 (PABPC3). In our screens, PABPC1 (NP_002559) was identified and confirmed as a direct binding partner of TTP, even in cellular lysates depleted of RNA by RNAse digestion. PABP apparently bound to the TZF domain of TTP, using its C-terminal region, in both the presence and absence of co-transfected ARE-containing RNA. Interestingly, certain non-RNA binding mutants of TTP could still interact with PABP in co-immunoprecipitation experiments, despite the fact that the TZF domain is thought to be the RNA binding domain. PABP is a nucleocytoplasmic shuttling protein [63] that can function both as inhibitor of mRNA deadenylation and as an enhancer of translation by simultaneously binding to poly(A) tails of cellular mRNAs and to the eukaryotic translation initiation factor 4G (eIF4G). This interaction allows for the formation of circular mRNA loops by providing sites of interaction for PABP and eIF4E [64], [65], and ultimately promotes both mRNA stability and protein translation. These studies suggest that TTP not only functions in the mRNA deadenylation and decay processes previously known to be affected by PABP, but may also be involved in the mRNA translation events influenced by PABP. As suggested previously by Wilusz et al., 2001 [66] it is possible that a physical interaction between TTP, already bound to its target mRNA, and PABP, could lead to dissociation of PABP from the poly(A) tail, making the tail more accessible to 3′-5′ exonucleases and thus accelerated decay.

In the two hybrid screens, two fragments of hTTP (aa 150–325 and aa 223–326) from the C-terminal region pulled out several partial prey clones of CIN85, corresponding to the N-terminal fragments aa 4–264 and aa 4–450. The interaction of human TTP with CIN85 was validated in co-immunoprecipitation experiments in HEK293 cells. Binding of full-length CIN85 to TTP required an intact C-terminal PXXXPR motif in the TTP protein, a motif shown to interact with the N-terminal SH3 domain of CIN85. Surprisingly, mouse TTP (mTTP), as well as other human and mouse members of the TTP family (hZFP36L1, hZFP36L2, and mZFP36L3), all lack this sequence. They also failed to bind to CIN85 in the two-hybrid screen or in the co-immunoprecipitation assays. However, when we replaced the threonine in the corresponding sequence in mTTP with proline (T302P), to recreate the human PXXXPR motif, it permitted the binding of mTTP to CIN85. Moreover, changing the first proline in this motif to valine (P309V) in hTTP eliminated the binding of hTTP to CIN85. These results suggested that CIN85 binding is specific to human TTP among the human TTP family members, and does not occur normally in the mouse. However, the PXXXPR binding motif is present in TTP in all other mammals tested, including rat.

In this study, binding of CIN85 to hTTP did not appear to alter the binding of hTTP to an ARE-containing RNA probe, nor did it alter the effect of TTP on the stability of a TNF-based mRNA probe. One possibility is that binding of TTP to CIN85 may affect the function of CIN85. For example, CIN85 can regulate the activity of the protein kinase MEKK4, by alleviating auto-inhibition and permitting auto-phosphorylation, resulting ultimately in the activation of protein kinase pathways such as the p38 MAP kinase pathway, involving MKK3, MKK4 and MKK6, and the JNK pathway, through MKK7 or MKK4 [21], [67], [68]. MKK4 and MKK7 can phosphorylate and activate JNK, whereas MKK3 and MKK6 can phosphorylate and activate the p38 MAPK [67]. Through these pathways and others, MEKK4 is a major mediator of oxidative and environmental stress such as osmotic shock, UV irradiation, wounding, and exposure to inflammatory factors [21], [67], [68], [69]. MEKK4 can also bind to TNF-receptor-associated factor (TRAF4) in TNF receptor signaling cascades, and to MAPK in cytokine signaling [67]. CIN85 can also activate other kinases, including Src family kinase-Cbl, activated receptor tyrosine kinases (RTKs), and the p85α subunit of phosphatidylinositol 3-kinase (PI3-K p85α) [18], [20], [70], [71], [72], [73].

One interesting finding from this study is that the interaction of hTTP with CIN85 led to an increase in TTP phosphorylation; specific residues whose phosphorylation was increased following CIN85 binding included Ser 66 and Ser 93. Strikingly, the electrophoretic shift resulting from this phosphorylation occurred when CIN85 binding activity was restored in mTTP, with the T302P mutation, and the electrophoretic shift was lost from hTTP after mutating the CIN85 binding site. This increase in hTTP phosphorylation upon CIN85 binding led us to investigate the possibility that a CIN85-activated protein kinase might be involved in a three-way complex with hTTP. This was indeed the case, since both hTTP and MEKK4 appeared to associate with CIN85 in separate two-protein complexes, and hTTP was found to associate with MEKK4 in the absence of exogenous CIN85. The three proteins together appeared to form a three-protein complex. Interestingly, the interaction of hTTP with MEKK4 in the absence of CIN85 did not appear to lead to the same enhanced phosphorylation of hTTP, suggesting that CIN85 binding is required for these phosphorylation events to occur.

Binding of hTTP by CIN85 may increase the phosphorylation of hTTP by several possible mechanisms, including (1) altering the conformational structure of TTP to make it a better kinase substrate; or (2) recruiting protein kinases to the vicinity such as MEKK4. This mechanism remains to be worked out. Also unclear is the effect of this binding and increased phosphorylation of hTTP on its activity or function. In our assays, the hyperphosphorylated hTTP appeared to bind relatively normally to an ARE-containing RNA probe, and to promote normally the destabilization of a TNF transcript-based, ARE-containing RNA probe in the co-transfection assays. These relatively crude assays cannot rule out minor effects on quantitative aspects of these hTTP activities. It may be that the hyper-phosphorylation of hTTP at these serine residues affects hTTP's intrinsic stability in cells, or its interactions with other proteins. These and other possibilities will require further investigation.

## Materials and Methods

### Yeast Two Hybrid Screening

Automated two-hybrid screening using ProNet technology was performed by Myriad Genetics, Salt Lake City, UT, as previously described [74], [75]. To construct “bait” plasmids expressing hTTP or its fragments fused to the yeast Gal4 DNA binding domain, fragments of hTTP of approximately 150 to 300 base pairs in length that spanned the entire protein coding region of hTTP were amplified by PCR, and were transformed together with Gal4 DNA binding domain vector DNA into a yeast strain with a mating-type locus designated MAT (*MAT*α *trp1-901 leu2-3*,*112 ura3-52 his3-200 ade2 gal4Δ gal80*) and selected on -Trp plates. Self-activating clones were identified by mating with empty activation domain vector in a MATα strain (MATα trp1-901 leu2-3,112 ura3-52 his3-200 gal4Δgal80 LYS2:GAL-HIS3 GAL2-ADE2 met2::GAL7-lacZ) and were eliminated in the process.

Several different “prey” libraries encoding potential TTP-interacting proteins were used. These were constructed from poly(A)+ RNA derived from the following sources: Mixed human breast cancer and prostate cancer cell lines, in a library containing approximately 80 million clones; a normal human spleen library of 11 million clones; and a normal human brain tissue library of 60 million clones. In each case the library was cloned downstream of the Gal4 activation domain (residues 768–881). MATα baits were mated with MATα prey and selected on -Trp, -Leu, -His, -Ade plates. His and Ade selections were used to isolate bait/prey interactions. DNA extracted from yeast colonies was used to transform *E. coli*, and bait plasmids were recovered through kanamycin selection and prey plasmids by ampicillin selection. Plasmids were re-transformed into yeast, and interactions were confirmed by liquid β-galactosidase assays. The prey clones were identified by DNA sequencing. DNA encoding an ARE-containing RNA derived from TNF ARE RNA (bp 1341–1364 of GenBank accession number NM_000594.2) was cloned downstream of ADH and CYC1 promoters in a plasmid with a URA3 auxotrophic marker gene. Yeast carrying “bait” and RNA expression plasmids were selected on –Trp, -Ura plates and screened as above.

### Plasmid Constructs

The epitope tag derived from influenza virus hemaglutinin protein [76] was fused to the last amino acid of hTTP cDNA by the PCR primer-overlapping mutagenesis technique and subcloned into the *HindIII* site of vector CMV.BGH3′/pBS+ to generate HA-hTTP as described [8]. Full-length cDNAs for human CIN85, ZFP36L1, and ZFP36L2 were sub-cloned into CMV.BGH3′/BS+ and modified with amino-terminal RGS-6His-tags and carboxyl-terminal epitope tags, either HA or FLAG, by insertion of oligonucleotide linkers into *Hind*III and *Apa*I digested CMV.BGH3′/BS+ to create vectors pCMV-FLAG-BGH3′ and pCMV-HA-BGH3′, respectively. Restriction endonuclease *BamH1* and *Xba*I sites were inserted into pCMV-BGH3′. A cDNA clone for CIN85 (clone ID 3906722) was obtained from the I.M.A.G.E. consortium through Open Biosystems (Huntsville, AL). The mouse ZFP36L3 (pFlag-muL3) has been described [17]. Expression constructs of HA or FlagTagged Poly-A binding protein (PABP) (GeneBank accession number BC015958) were created by RT-PCR using total cellular RNA from HeLa cells (ATCC catalog number CCL-2) as a template for reverse transcription and were cloned into the *Asp718* and *XbaI* restriction sites of CMV.BGH3′/BS+. Expression plasmids for mouse HA-TTP and the plasmid construct CMV.mTNF-α have been described [8]. The C-terminal deletion expression constructs of HA-hTTP, namely, HA-hTTP 1-322, HA-hTTP 1-319, HA-hTTP 1-313, and HA-hTTP 1-290, were kindly provided by Dr. Wi S. Lai in our laboratory and were similarly generated by PCR using human WT HA-hTTP as a template and sub-cloned into the *HindIII* site of the vector CMV.BGH3′/pBS+ as described above [8]. Expression plasmids HA-hTTP/P309V and HA-mTTP/T302P were generated by using WT HA-hTTP and WT HA-mTTP respectively, in a kit from QuickChange Site-Directed Mutagenesis (Stratagene, La Jolla, CA). Correct sequences of all plasmid inserts were confirmed by dRhodamine Terminator Cycle Sequencing (Perkin-Elmer Life Sciences, Boston, MA).

An HA-MEKK4 expression plasmid was a gift from Dr. Gary Johnson, University of North Carolina at Chapel Hill, NC, and has been described [77]. Expression plasmids for Flag-CIN85 and its deletion constructs have been described [18] and were gifts from Dr. Sachiko Kajigaya, National Heart, Lung and Blood Institute, National Institutes of Health, Bethesda, MD. The expression plasmid HA-MARCKS (pBS-CMV/H80K-HA) was constructed by subcloning a 1.04 kb *ECOR1/HindIII* cDNA fragment of human MARCKS containing the entire protein coding region with an attached HA-tag into the *ECOR1/HindIII* sites of pBS-CMV. The glyceraldehyde-3-phosphate dehydrogenase (GAPDH) cDNA has been described [40].

### Cell Transfections, Immunoprecipitations and Western Blotting

HEK 293 cells (ATCC catalog number CRL-1573) were maintained in Minimum Essential Medium (MEM; Invitrogen) supplemented with 10% fetal bovine serum, 100 U/ml penicillin and 100 µg/ml streptomycin. Transient transfection was performed using a standard CaPO_4_ procedure as described [8]. Briefly, 0.2 – 0.5 µg of plasmid DNA was transfected together with carrier pBluescribe SK- (pBS) DNA to make a total of 5 µg per 100 mm dish. Sixteen h after the addition of DNA, cells were washed twice with MEM at 37°C, and replenished with fresh complete medium.

After a further 24 h of incubation, cells were washed twice with ice-cold phosphate-buffered saline (PBS), and all liquid was removed by aspiration. Cells were lysed by direct addition to the culture dish of 600 µl/10 cm dish of one of two buffers. The first was a radioimmunoprecipitation assay (RIPA) buffer (150 mM NaCl, 1% (v/v) nonidet P-40 (NP-40), 0.5% (w/v) sodium deoxycholate, 0.1% (w/v) sodium dodecyl sulfate, 50 mM Tris-HCl, pH 7.5) supplemented with protease inhibitors (0.2 µg/ml leupeptin, 0.2 µg/ml pepstatin and 0.5 mM 4-(2-Aminoethyl) benzenesulphonyl fluoride (ICN Biochemicals, Costa Mesa, CA)). Cell debris and buffer were scraped from the plate on ice and extracted for a further 30 min by tumbling at 4°C. Extracts were clarified by centrifugation at 100,000×g for 45 min at 4°C. NP-40 extracts were prepared as follows: Cells were scraped from 10 cm dishes, combined and sedimented at 600×g for 3 min at room temperature. PBS was aspirated and cells were gently resuspended in a second buffer, NP-40 hypotonic lysis buffer (0.2% (v/v) NP-40, 10 mM KCl, 3 mM MgCl_2_, 10 mM Hepes-NaOH, pH 7.6) supplemented with protease inhibitors as above, and incubated on ice for 15 min. Complete lysis was confirmed by light microscopy of cells exposed to trypan blue. Extracts were clarified by centrifugation at 22,000×g for 15 min at 4°C, the KCl concentration was adjusted to 50 mM, and glycerol was added to a final concentration of 10% (v/v). All extracts used in immunopurification assays were treated with RNase. None of the extracts used in this study was frozen prior to immunoprecipitation. Remaining extracts were stored at –80°C for immunoblotting.

For immunoprecipitation, 1 mg of cellular protein in 1 ml of extract in RIPA buffer was incubated at 4°C with 4 µg (20 µl) anti-FLAG (Sigma, St. Louis, MO) or anti-HA (F-7, Santa Cruz Biotechnology, Santa Cruz, CA) monoclonal antibody overnight, and then added to 100 µl (packed volume) protein A Sepharose 4B beads (Pharmacia, Uppsala, Sweden) and mixed on a rotator (BD, Franklin Lakes, NJ) for 3 to 4 h at 4°C. Beads were washed by centrifugation at 1000×g for 1 min 3 times with 1 ml of lysis buffer. SDS sample buffer was added directly to the beads.

Western blotting was performed using 50 µg of protein in cellular extracts mixed with a 1/5 volume of 5× SDS sample buffer, boiled for 3 min, and then loaded onto SDS–10% PAGE gels. Western blotting was performed by standard techniques. Membranes were incubated in Tris-buffered saline–0.5% Tween 20 with either polyclonal antiserum HA.11 (1∶2,500), or with anti-FLAG antibodies directly coupled to horseradish peroxidase, as appropriate. In some instances, other rabbit polyclonal antibodies were used, including anti-nucleolin (C23, Santa Cruz Biotech. Inc., Santa Cruz, CA), anti-HSP70 (K-20 from Santa Cruz) or anti-CIN85 (HQ-17, Sigma). Incubation of membranes with secondary antibodies and development were as described elsewhere [39].

### Immunofluorescence and Confocal Microscopy

For immunostaining, cells were cultured on glass cover slips (Ted Pella Inc; Redding, CA) and transfected with the FLAG- or HA-tagged constructs using the Fugene 6 reagent (Roche Applied Science). Two days after transfection, cells were washed twice with PBS and fixed with 3.7% (v/w) formaldehyde for 10 min at room temperature, permeabilized with 0.5% (v/v)Triton X-100, and stained with mouse anti-FLAG monoclonal antibody M2 (Sigma) and rabbit anti-HA polyclonal antibody Y-11 (Santa Cruz Biotechnology), followed by fluorescein isothiocyanate-labeled goat anti-mouse secondary antibody Alexa Fluor 488, or rhodamine-conjugated goat anti-rabbit secondary antibody Alexa Fluor 594 (Invitrogen), as appropriate. The stained cells were mounted with DAPI-VECTASHIELD mounting medium with 4′, 6-diamidino-2-phenylindole (Vector Laboratories) to visualize the nuclei. Stained proteins were visualized by confocal microscopy using a FluoView™ laser scanning epi-fluorescence Olympus FV1000 microscope.

### Northern Blotting

For northern analysis, HEK 293 cells were transfected as described above. Twenty-four hours after the removal of the transfection mixture, total cellular RNA was harvested using the illustra RNAspin mini RNA isolation kit (GE Healthcare, Buckinghamshire, UK). Northern blots were prepared as described elsewhere [8]. Blots were hybridized to a randomly primed, α-^32^P-labeled mTTP cDNA [8] or a ∼1-kb *Nar*I-*Bgl*II fragment of mTNF cDNA. Some blots were also hybridized with an α-^32^P-labeled glyceraldehyde-3-phosphate dehydrogenase (GAPDH) cDNA probe [40] or with a 570-bp PCR product (nucleotides 634–1203; GenBank accession number NM_031892) of a CIN85 cDNA [18].

### RNA Electrophoretic Mobility Shift Assay

Cytosolic extracts (2 µg of protein) prepared from HEK 293 cells transfected with vector alone, or with expression constructs driven by the CMV promoter, were incubated with 0.6 ng of 5′ biotin-labeled human TNF-ARE probe (Invitrogen Corp, CA) at room temperature for 20 min in 20 µl of lysis buffer (without protease inhibitors) containing 10 mM Hepes (pH 7.6), 40 mM KCl, 2.5% (v/v) glycerol and 3 mM MgCl_2_. Heparin and yeast tRNA were added to final concentrations of 2.5 µg/µl and 50 ng/µl, respectively, for an additional 10 min. RNA not associated with protein was digested with 100 U of RNase T_1_ for 20 min at room temperature; the reaction mixture was then loaded onto a 6% nondenaturing acrylamide gel and subjected to electrophoresis at 160 V for 90 min, in 0.4 X Tris-borate-EDTA buffer. Gels were transferred to Biodyne B nylon membranes (0.45 µm) (Thermo Scientific, IL) in 0.4 X Tris-borate-EDTA buffer at 80 V for 1 hr. Unbound probe and RNA-protein bound signals were detected using a stabilized streptavidin-HRP conjugate antibody in a chemiluminescent nucleic acid detection module kit (Thermo Scientific, IL), per the manufacturer's instructions, and exposed to BIOMAX MR films from Kodak.

### Dephosphorylation of hTTP

Some samples of hTTP lysates overexpressed in HEK 293 cells were dephosphorylated with calf intestinal alkaline phosphotase (CIAP) (Invitrogen Corp., Carlsbad, CA) as described previously [35].

### Analysis of Phosphorylation Sites in hTTP

For in gel digestion of proteins, phosphorylation sites from eluted samples of hTTP from HEK-293 cells overexpressing Flag-hTTP alone, or Flag-hTTP and HA-CIN85 together, were analyzed by performing MALDI-MS and ESI-MS on hTTP tryptic peptides that were extracted from the polyacrylamide gels. In brief, clarified supernatants of cell lysates in RIPA buffer were subjected to a Flag-peptide affinity column, and hTTP was eluted using 3XFlag peptides as per the manufacturer's instructions (Sigma). Gel bands were excised manually and digested with trypsin (Promega) for 8 h in an automated fashion with a Progest robotic digester from Genomic Solutions. Resulting peptides were lyophilized and then resuspended in 35 µl of 0.1% formic acid.

For phosphopeptide enrichment, TiO_2_ tips (Glygen) were employed using essentially the manufacturer's recommended protocol.

A variety of MS and affinity techniques were employed in efforts to identify sites of phosphorylation on hTTP. ESI-MS (see below) was performed on hTTP peptides that were from solution digests or extracted from polyacrylamide gels with and without MOAC enrichment. Candidate phosphorylated peptides determined by automated database searching were manually validated. Once sites were identified, nanoLC-ESI-MS experiments were performed on hTTP digests in efforts to obtain information on the relative extent of phosphorylation at the identified site using approaches essentially as described [78].

NanoLC-ESI-MS and MS/MS analyses were performed on hTTP digests using an Agilent 1100 nanoLC system on-line with an Agilent 6340 ion trap mass spectrometer with the Chip Cube Interface. Briefly, 20 µl of hTTP digest were loaded onto an Agilent C_18_ chip (75 µm×43 mm) followed by a 15 min wash of 5% acetonitrile, 0.1% formic acid. Peptides were eluted by applying a linear gradient from 5% acetonitrile, 0.1% formic acid to 50% acetonitrile, 0.1% formic acid to the column over 45 min. This was followed by a 5 minute gradient from 50% acetonitrile, 0.1% formic acid to 95% acetonitrile, 0.1% formic acid and then a 10 minute hold at 95% acetonitrile, 0.1% formic acid. The mass spectrometer was used in the positive ion, standard enhanced mode and included settings of a mass range from 200 to 2200 m/z, an ionization potential of 2.1 kV, an ICC smart target of 100000 ions accumulated in the trap or 200 milliseconds of accumulation, and a 1.0 volt fragmentation amplitude. MS/MS data were acquired using a data dependent acquisition format, with the six most abundant ions from each MS scan further interrogated by MS/MS. The automated switching for MS/MS required a threshold of 5000 counts.

For automated database searching, peak lists were generated from the data obtained from each nanoLC-ESI-MS/MS analysis using the Data Extractor feature of the SpectrumMill software from Agilent. The resulting extracted data were then searched against the NCBI non-redundant database using the MS/MS Search function in the SpectrumMill software. Search settings included enzyme specificity with up to two missed cleavages allowed, a precursor ion mass tolerance of 1.5 Da, a product ion mass tolerance of 1.0 Da, variable methionine oxidation, serine, threonine, and tyrosine phosphorylation, and a minimum matched spectral intensity of 70%. Sequence assignments of MS/MS spectra were manually validated.
